# Assessing the reliability of CBCT-based AI-generated STL files in diagnosing osseous changes of the mandibular condyle: a comparative study with ground truth diagnosis

**DOI:** 10.1259/dmfr.20230141

**Published:** 2023-09-04

**Authors:** Kaan Orhan, Alex Sanders, Gürkan Ünsal, Matvey Ezhov, Melis Mısırlı, Maxim Gusarev, Murat İçen, Mamat Shamshiev, Gaye Keser, Filiz Namdar Pekiner, Maria Golitsyna, Merve Önder, David Manulis, Cemal Atakan

**Affiliations:** 1 Faculty of Dentistry, Department of Dentomaxillofacial Radiology, Ankara University, Ankara, Turkey; 2 Diagnocat Inc, West Sacramento, California, United States; 3 Faculty of Dentistry, Department of Dentomaxillofacial Radiology, Near East University, Nicosia, Cyprus; 4 Faculty of Dentistry, Department of Dentomaxillofacial Radiology, International Final University, Nicosia, Cyprus; 5 Faculty of Dentistry, Department of Dentomaxillofacial Radiology, Nevşehir Hacı Bektaş Veli University, Nevsehir, Turkey; 6 Faculty of Dentistry, Department of Dentomaxillofacial Radiology, Marmara University, Istanbul, Turkey; 7 Faculty of Science and Letters, Department of Statistics, Ankara University, Ankara, Turkey

**Keywords:** temporomandibular joint, mandibular condyle, stereolithography, cone-beam computed tomography, artificial intelligence

## Abstract

**Objectives:**

This study aims to evaluate the reliability of AI-generated STL files in diagnosing osseous changes of the mandibular condyle and compare them to a ground truth (GT) diagnosis made by six radiologists.

**Methods:**

A total of 432 retrospective CBCT images from four universities were evaluated by six dentomaxillofacial radiologists who identified osseous changes such as flattening, erosion, osteophyte formation, bifid condyle formation, and osteosclerosis. All images were evaluated by each radiologist blindly and recorded on a spreadsheet. All evaluations were compared and for the disagreements, a consensus meeting was held online to create a uniform GT diagnosis spreadsheet. A web-based dental AI software was used to generate STL files of the CBCT images, which were then evaluated by two dentomaxillofacial radiologists. The new observer, GT, was compared to this new STL file evaluation, and the interclass correlation (ICC) value was calculated for each pathology.

**Results:**

Out of the 864 condyles assessed, the ground truth diagnosis identified 372 cases of flattening, 185 cases of erosion, 70 cases of osteophyte formation, 117 cases of osteosclerosis, and 15 cases of bifid condyle formation. The ICC values for flattening, erosion, osteophyte formation, osteosclerosis, and bifid condyle formation were 1.000, 0.782, 1.000, 0.000, and 1.000, respectively, when comparing diagnoses made using STL files with the ground truth.

**Conclusions:**

AI-generated STL files are reliable in diagnosing bifid condyle formation, osteophyte formation, and flattening of the condyle. However, the diagnosis of osteosclerosis using AI-generated STL files is not reliable, and the accuracy of diagnosis is affected by the erosion grade.

## Introduction

### Temporomandibular joint degeneration

Temporomandibular joint (TMJ) degeneration, also known as TMJ degenerative disease, is characterized by the structural and functional degradation of the TMJ. Common signs of this degenerative process may include discomfort, difficulty opening or closing the mouth, popping or clicking sounds when opening the jaw and even trismus. TMJ degenerative disease can be caused by various factors, including osteoarthritis, trauma, jaw misalignment, and excessive wear and pressure on the joint.^1^ For the diagnosis of TMJ osteoarthritis, cone-beam computed tomography (CBCT) and multi-detector computed tomography (MDCT) are considered superior to magnetic resonance imaging (MRI) in evaluating osseous changes.^2–4^ Although Hintze et al found no significant differences in the detection of morphological changes between CBCT and MDCT, the lower radiation dose makes CBCT preferable for diagnosis.^5,6^

The common morphological deformities and osseous changes of the condyle that can be analyzed by CBCT are as follows:^5,7,8^

- Flattening of the TMJ condyle surface
- Erosion of the TMJ condyle
- Bifid Condyle Formation
- Osteophyte Formation on the TMJ condyle
- Osteosclerosis of the TMJ condyle
- Hyperplasia of the TMJ condyle
- Hypoplasia of the TMJ condyle

### TMJ and CBCT

The SEDENTEX-CT project’s study results^9^ have prompted the European Academy of Dentomaxillofacial Radiology (EADMFR) to address the use of CBCT for the dentomaxillofacial area. In their publication titled 'Basic Principles for Use of Dental Cone-Beam CT: Consensus Guidelines of the EADMFR' in 2009, the EADMFR stated that CBCT offers numerous advantages over other 3D imaging modalities. These advantages include high resolution, lower patient exposure doses, and improved diagnostics for developmental anomalies, traumas, osteoarthritis, and ankylosis.^9,10^ CBCT also provides superior imaging of bony changes in the TMJ due to its higher resolution cross-sectional images and lower cost of examination equipment and facility^5^ However, CBCT is not reliable for interpreting soft tissues as it has lower image contrast compared to CT.^3^ Additionally, CBCT exhibits higher image noise than CT.^5^ While it is sometimes suggested to use a silicone index for TMJ imaging with CBCT, a specific patient preparation method is not mandatory.^5,11^ No specific contraindications have been reported yet, further establishing it as a favorable imaging method for the mandibular condyle.^5^

### Knowledge among dental practitioners on the interpretation of CBCT

According to the EADMFR, the level of knowledge among dentists regarding the interpretation of CBCT images may not always be sufficient, as dental schools may not provide enough lectures on this topic for undergraduate students. Moreover, dentomaxillofacial radiology post-graduate programs are not common in most European countries, making it difficult to consult 3D images with dental radiology specialists (oral diagnosis and dentomaxillofacial radiology specialists). Since most CT and CBCT images demonstrate the bony structures of the TMJ in larger field of view (FOV) values, the mandibular condylar process is a commonly observed anatomical structure in these 3D images.

### Digital Imaging and Communications in Medicine and Standard Triangle Language

Digital Imaging and Communications in Medicine (DICOM) and Standard Triangle Language (STL) are distinct data formats that exhibit interaction as the dental field increasingly adopts three-dimensional advancements. Understanding these standards' origins may be useful for comprehending the functions they perform. The first data format, known as DICOM (Digital Imaging and Communications in Medicine), has been the standard for medical digital radiography for many years. It encompasses not only the formats used for digital medical image archiving but also includes important communication protocols for diagnostic imaging processes. The primary purpose of the DICOM standard for digital radiographs is to ensure consistency in image file format. While the utilization of DICOM protocol requirements is common in larger centers such as dentistry faculties and hospitals, which need to perform, store, and manage various digital radiography, it is less practical for regular dental clinics. In a dental office, traditional CBCT scans are typically stored in DICOM format by imaging software. The STL format has gained significance in dentistry, particularly due to its association with 3D printing, computer-aided design, and computer-aided manufacturing. It represents the surface geometry of a three-dimensional object and has become the preferred data format for most 3D printers and milling systems. Unlike the DICOM approach which divides the volume into slices, the STL format breaks down the surface of the volume into triangular “tiles.” Consequently, the DICOM file tends to provide more information about the internal structure of the volume, whereas the STL file focuses on providing detailed information about the surface of the volume.”^10,12–15^

### AI-generated STL files

Artificial intelligence (AI) has transformed a variety of medical specialties, including radiology, over the past decade. The creation of three-dimensional models from medical imaging data, such as computed tomography (CT) scans, is one interesting area of use for AI in radiology. These generated STL models offer comprehensive representations of anatomical features, enabling improved examination and analysis. However, it is essential to assess the correctness and dependability of these AI-generated STL files before using them in the clinical routine.^16,17^

This study aims to evaluate the reliability of AI-generated STL files in diagnosing osseous changes of the mandibular condyle and compare them to a “ground truth” diagnosis made by six radiologists.

## Methods and materials

The study protocol was approved by the Health Sciences Ethics Committee on July 28, 2022, with the file number YDU/2022/105–1591. The Helsinki Declaration’s guiding principles were used in the investigation. Deidentification was performed in line with the Information Commissioner’s Anonymization: managing data protection risk code of practice (https://ico.org.uk/media/1061/anonymisation-code.pdf) and validated by the aforementioned institution. The “Anonymisation: managing data protection risk code of practice” guides anonymizing personal data to protect individuals' privacy. It explains legal concepts, techniques, and risks related to anonymization. The code emphasizes the benefits of effective anonymization, supports privacy by design principles, and promotes transparency. It applies to organizations converting personal data into anonymized data and helps them comply with data protection laws while making data available for research or publication. Compliance is not mandatory, but following the code reduces legal risks and improves public trust in data handling. Only deidentified anonymized retrospective data were used for research, without the active involvement of patients. After obtaining the informed consent, patients whose condylar process is located within the field of view of a CBCT scan were included in the study. No specific clinical information was required.

A total of 432 anonymous retrospective CBCT images, which were acquired from four different universities and four different CBCT units, were evaluated in this study. The CBCT units that were used in this study are:

- Promax3D Mid (Planmeca; Helsinki, Finland), 90 kV, 10 mA, FOV = 16x 16cm, Voxel size = 0.2 mm^3^
- NewTom 3G (NewTom; Verona, Italy), 120 kVp, 3-5mA, FOV = 20x 25cm, Voxel size = 0.3 mm^3^
- Veraviewepocs 3D R100/F40 (Morita; Kyoto, Japan), 90 kVp, 5mA, FOV = 4x 8cm, Voxel size = 0,125 mm^3^
- Sirona Orthophos SL 3D (Dentsply Sirona; Bensheim, Germany), 90kVp, 3–16 mA, FOV = 11x 10cm, Voxel size = 0,08 mm^3^

Six dentomaxillofacial radiologists independently evaluated the data for flattening, erosion, osteophyte formation, osteosclerosis, bifid condyle formation, and other conditions such as hyperplasia and hypoplasia. The evaluation was conducted using anonymized DICOM files shared by each author through a cloud system. Each radiologist assessed the data individually and documented their findings on a spreadsheet.

Flattening was evaluated as the absence (Grade 0) or presence (Grade 1) of condyle flattening. It is important to note that although we included flattening as an osseous change of the TMJ, it is not considered a degenerative joint disease by the International RDC/TMD Consortium Network and Orofacial Pain Special Interest Group.^1^ Erosion was evaluated in four different grades: the absence of erosion (Grade 0), reduced density localized only at cortical plates (Grade 1), presence of pitted and irregular contours extending into the superior layers of adjacent subcortical bone (Grade 2), and presence of pitted and irregular contours extending to the inferior portion of the superior layers of adjacent subcortical bone (Grade 3). Osteophyte formation was evaluated in four different grades: absence of osteophyte formation (Grade 0), marginal bony prominence above the condyle shorter than 1 mm (Grade 1), marginal bony prominence above the condyle between 1 and 2 mm (Grade 2), and marginal bony prominence above the condyle longer than 2 mm (Grade 3). Osteosclerosis was evaluated in two different grades: absence (Grade 0) or presence (Grade 1) of osteosclerosis.

Following the end of the first evaluation stage, all observers recorded their evaluations on an online spreadsheet individually. A statistician gathered those records for statistical evaluation and a comparison was made according to the hypothesis of whether the “average measures” corresponding intraclass correlation coefficient (ICC) value is 0. If the obtained significance value (*p*-value) is less than 0.05, the hypothesis is rejected, and it can be concluded that the ICC value is significant. ICCs were classified as: 0.00–0.50: poor reliability, 0.50–0.75: moderate reliability, 0.75–0.90: good reliability, and 0.90–1.00: excellent reliability. A consensus meeting was set to discuss the differences in diagnoses and to create a uniform ground truth observer (GT). Following the online meeting, all observers reached a consensus, and it was also recorded on a spreadsheet file as observer GT.

**Figure 1.**
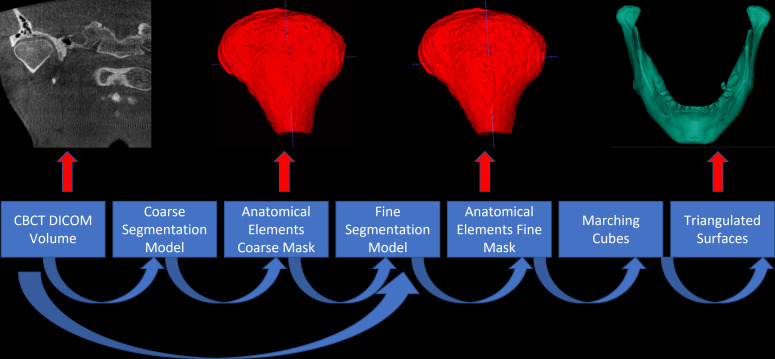
Inference algorithm of the present study.

STL files were generated from the CBCT images that were previously evaluated by the observers using a web-based dental AI software (**Diagnocat Inc., San Francisco, CA, USA**). The model’s approach to handling large volume sizes involves a coarse-to-fine strategy, where inference is performed at progressively finer scales guided by results from coarser stages. To handle large volume sizes, the software adopts a coarse-to-fine approach where inference is performed at progressively finer scales, guided by results from coarser stages. The two-stage model includes a coarse stage and a fine stage. The coarse stage analyzes the entire volume at a resolution of 1 mm, providing a preliminary segmentation. The fine stage refines the results using patch-based semantic segmentation at a voxel scale of 0.25 mm. Then, the data are normalized and split into training, development, and test sets. We use 90% for training, 5% for development, and 5% for testing. The coarse model is trained with soft targets, while the fine model is trained using patches. Coarse hints from the coarse model are interpolated and provided as additional input channels to the fine model. Both the coarse and fine stages employ the 3D U-Net architecture for semantic segmentation. Data augmentations and loss functions address the class imbalance. The algorithm was implemented using the Python U-Net implementation and trained on an NVIDIA GeForce RTX A100 GPU. During testing, the patch-based approach may produce lower quality predictions near the borders of output patches. To mitigate this issue, inference using overlapping patches was performed and the predictions were aggregated to give more weight to the center voxel of each output patch. The patches' overlap is set to 16 (Figure 1).

Automatic segmentation of the mandibular condyles was downloaded as STL files and re-evaluated by two dentomaxillofacial radiologists individually in 3D models, 3 months after the initial radiographic evaluation. The consensus of those two dentomaxillofacial radiologists is recorded on a spreadsheet file as observer STL. The diagnostic value of STL models was evaluated by ICC and κ values between observer GT and observer STL.

## Results

The condyles of the TMJ were evaluated for five osseous changes, including flattening, erosion, osteophyte formation, osteosclerosis, and bifid condyle formation, by six dentomaxillofacial radiologists. The Fleiss multirater κ values for these changes were 0.923, 0.923, 0.857, 0.608, and 0.756, respectively. The interclass correlation (ICC) values for these changes ranged from 0.927 to 0.971, indicating excellent reliability among the observers (Table 1).

**Table 1.** Intraclass Correlation Coefficient and Fleiss Multirater κ values for parameters among the observers

Comparison Among the Observers	**Intraclass Correlation Coefficient**	Fleiss Multirater κ	Significance
Osteophyte Formation	0,944	0,857	0,000
Erosion	0,968	0,923	0,000
Flattening	0,969	0,923	0,000
Bifid Condyle Formation	0,940	0,756	0,000
Osteosclerosis	0,927	0,608	0,000

**Figure 2.**
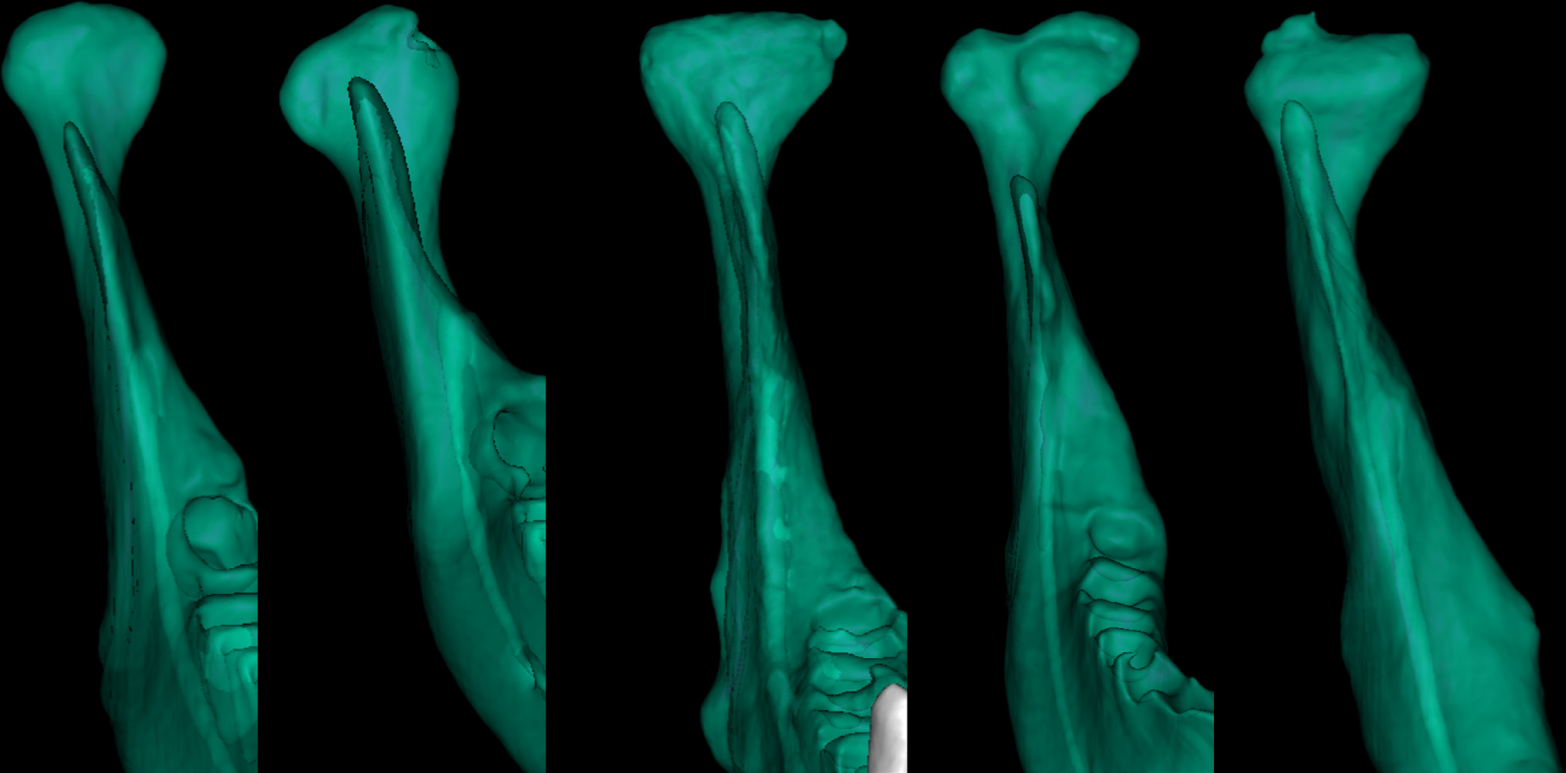
STL files of a healthy case with erosion, flattening, bifid condyle formation, and osteophyte formation greater than 2 mm.

To establish a consensus, a meeting was conducted to review the diagnoses, resulting in the generation of a ground truth (GT) observer. According to the ground truth observer’s spreadsheet, out of 864 condyles, 372 cases of flattening, 185 cases of erosion, 70 cases of osteophyte formation, 117 cases of osteosclerosis, and 15 cases of bifid condyle formation were identified (Figure 2).

When comparing the diagnoses based on the examination of STL files to those of the ground truth observer, the interclass correlation (ICC) values for flattening, erosion, osteophyte formation, osteosclerosis, and bifid condyle formation were 1.000, 0.782, 1.000, 0.000, and 1.000, respectively. The Fleiss Multirater κ values were 1.000, 0.312, 1.000, 0.000, and 1.000, respectively (Table 2).

**Table 2.** Intraclass Correlation Coefficient and Fleiss Multirater κ values for parameters between GT and STL observers

GT-STL Comparison	Intraclass Correlation Coefficient	Fleiss Multirater κ	Significance
Osteophyte Formation	1,000	1,000	0,000
Erosion	0,782	0,312	0,000
Flattening	1,000	1,000	0,000
Bifid Condyle Formation	1,000	1,000	0,000
Osteosclerosis	0,000	0,000	0,500

## Discussion

Applications for computer-aided design (CAD) and 3D printing can utilize STL files. CBCT scans can provide STL files that can be used to make 3D models of the TMJ in dentistry. The visualization of the joint’s anatomy and the detection of any anomalies can both benefit from the usage of STL files in the diagnosis of TMJ diseases. The following are some particular ways in which STL files might assist with the identification of TMJ issues on computed tomography scans:^3,10,13,14,18–20^

- Visualization of bony structures: STL files allow clinicians to view the bony structures of the TMJ in 3D, which can provide greater detail than 2D CT scans alone. This can aid in the identification of bony abnormalities such as condylar hyperplasia and erosion.
- Assessment of joint space: STL files can be used to measure the distance between the condyle and fossa, which can aid in the diagnosis of disc displacement or joint space narrowing.
- Evaluation of condylar position: STL files can help assess the position of the condyle in relation to the fossa, which can aid in the diagnosis of condylar displacement or subluxation.
- Planning of surgical or non-surgical interventions: STL files can be utilized to plan TMJ surgery or non-surgical therapies. For instance, a surgical guide 3D-printed from an STL file can help with the accurate implantation of orthopedic implants during TMJ surgery.

STL files generated from cone beam computed tomography (CBCT) scans can provide a 3D model of the TMJ, allowing for a comprehensive evaluation of bony structures, including the mandibular condyle, glenoid fossa, and articular eminence. Compared to traditional 2D imaging methods, such as panoramic and lateral cephalometric radiographs, CBCT scans and STL files provide more accurate and detailed information about the complex bony anatomy of the TMJ. However, it is important to note that the quality of the CBCT scan and the expertise of the operator in generating the STL files can greatly affect the accuracy and precision of the resulting 3D model. Additionally, STL files do not provide information on the soft tissue structures of the TMJ, such as the articular disc, which is important in the diagnosis and treatment of TMJ disorders. Therefore, while STL files can be a valuable tool in evaluating the bony features of the TMJ, they should be used in conjunction with other imaging methods and clinical findings to provide a comprehensive evaluation and diagnosis of TMJ disorders.^3,10,13,14,18,20–25^

The results of this study also reported perfect success for the bifid condyle formation, osteophyte formation, and flattening; however, the ICC values for erosion and osteosclerosis were 0.782 and 0.000, respectively. As the STL files that were generated by the AI software we used were unable to differentiate the cortical borders from the trabecular bone, the dentomaxillofacial radiologists who evaluated the STL files were unable to detect Grade 1 erosions. It is fair to state that advanced erosions are demonstrative in STL files, but the diagnosis of the initial erosions requires exact visualization of the cortical borders. Moreover, the specialists were unable to detect osteosclerosis of the trabecular bone of the condylar process in STL files, as the STL files only demonstrated superficial formation. In this study, none of the osteosclerotic condyles could be diagnosed in STL files.

There were five major limitations to this study. As all of the STL files were generated from 4 CBCT devices and a single AI software, this study cannot state whether the results will be the same for all other CBCT devices and software. A better generalizability study should be done with more CBCT devices and multiple software since Kamio et al^20^ reported that differences exist depending on the STL segmentation of the software. Also, STL files do not provide information on the soft tissue structures of the TMJ, such as the articular disc; thus, this study does not cover all of the diagnoses of TMJ disorders. Moreover, As STL files can only represent the outer features of a structure any evaluation that requires the differentiation of the trabecular and cortical bone may not be done with this technique. The study may not compare the accuracy of the AI-generated STL files with other imaging modalities, such as MDCT or MRI scans, which can limit the understanding of the overall accuracy and usefulness of STL files for TMJ diagnosis.

In their study, Kamio et al^20^ assessed the efficacy of nine different software programs for generating STL models from MDCT DICOM files. The researchers utilized a dry human mandible with two 10-mm-diameter bearing balls during the scanning process. The results indicated that there were variations in file size and the number of triangles comprising each STL model across all software packages, but there were no statistically significant differences observed. The mean shape error for the mandibular STL model was 0.11 mm, and there was no significant variation between the software packages. The authors emphasized the importance of understanding the features of each software package, particularly in the fine and thin regions of the osseous structures, despite the observed differences in performance.

## Conclusion

TMJ evaluation of the AI-generated STL files was perfect for bifid condyle formation, osteophyte formation, and flattening; however, osteosclerosis could not be diagnosed just using AI-generated STL files. The grade of erosion was a determining factor in the diagnoses, as Grade 1 erosions could not be diagnosed on STL files as the cortical bone and trabecular bone were inseparable from each other.
